# Biological evaluation of the toxicity and the cell cycle interruption by some benzimidazole derivatives

**DOI:** 10.1007/s13277-016-4828-1

**Published:** 2016-03-01

**Authors:** Katarzyna Błaszczak-Świątkiewicz, Joanna Sikora, Jacek Szymański, Marian Danilewicz, Elżbieta Mikiciuk-Olasik

**Affiliations:** 1 Department of Pharmacy, Medical University, Muszynskiego 1, 90-151 Lodz, Poland; 2 Laboratory of Bioanalysis, Department of Pharmaceutical Chemistry, Drug Analysis and Radiopharmacy, Medical University, Muszynskiego 1, 90-151 Lodz, Poland; 3 Central Scientific Laboratory, Medical University, Mazowiecka 6/8, 92-215 Lodz, Poland; 4 Educational Center of the Medical University of Lodz, Pomorska 251, 92-213 Lodz, Poland; 5 Department of Pharmaceutical Chemistry, Drug Analysis and Radiopharmacy, Medical University, Muszynskiego 1, 90-151 Lodz, Poland

**Keywords:** Anticancer activity, Benzimidazole derivatives, Bioreductive agents, Cell cycle of benzimidazole, Toxicity of benzimidazole

## Abstract

In this work, the in vitro tests of biological activity of benzimidazoles were conducted. This group of benzimidazole derivatives was evaluated as potential bioreductive agents and their characteristic pro-apoptosis activity and cell cycle interruption on the human lung adenocarcinoma A549 cells were discussed. Their toxicity on the healthy human erythrocytes and their influence on the healthy human erythrocytes acetylcholinesterase enzyme (AChE) were established. Their apoptosis activity on A549 cells line was determined by Annexin V-APC test, and it was visualized by Hoechst test. In the next stage, their influence on the cell cycle interruption was determined by using the ribonuclease reagent. The AChE inhibition test was defined by the Ellman method, and the red blood cell lysis was defined by erythrotoxicity test. The results proved the pro-apoptosis properties of all tested compounds in normoxia and hypoxia. The DNA content assay showed that the benzimidazoles possess the ability to interrupt S phase of tumor cell cycle. The best activity in this action was presented by compound **1**, especially in hypoxia, and it proves that the *N*-oxide analogs are predispositioned to the hypoxic target. In this study, the benzimidazoles were found as potentially biocompatible and their inhibition of acetylcholinesterase was lower than tirapazamine and much lower than tacrine which constitutes their desired effect of potential biological activity.

## Introduction

The cancer ethiopathogenesis research plays a dominant role in the global scientific trends. The focus is on the bad epidemiological and demographic data concerning the increasing mortality because of the malignant neoplasms. The most common globally recorded cancers are the lung cancer in men and breast cancer in women [1, 2]. Still, the widely available methods of cancer treatment are surgery, therapy with non-selective chemotherapeutic agents, and radiotherapy or hormonal therapy [3]. While the last listed method is strictly based on the environmental change in tumor tissue, the cytostatic chemotherapy agents or cytotoxic drugs generally do not possess the selective antitumor activity. Most of them affect cell proliferation and can damage normal tissue as well as, especially unchanged, rapidly growing cells such as the bone marrow, epithelial cells, gametocytes, or lymphatic cells. The similar toxic effect on healthy tissue is exhibited by the most popular methods of radiotherapy [4]. Systemic toxicity is one of the major shortcomings of previously conducted anticancer therapies. Therefore, an effective anticancer therapy should bear the properties of a relatively safe therapy which will be revealed not only by radiotherapy and chemotherapy dosages, but it must primarily take into account knowledge of the effect of the compounds on the cell cycle. With assistance at searching the way for selectivity therapy came the progress of the bioreductive drugs [5]. The bioreductive therapy uses hypoxia as the target for main differences between normal and cancer cells [6]. Anticancer drugs because of their effect on cell division are divided into non-specific, specific for the cycle and specific for the phase. The last class of potential anticancer agents includes bioreductive agents with tirapazamine as the most important representative of this class of compounds [7–11]. The distinguishing characteristic feature of these compounds from the group of cytotoxic substances is their selectivity for hypoxic cells which in turn provides an opportunity to reduce the systemic toxic effects.

The study conducted by our team provided the initial evaluation of the toxicity of new benzimidazole derivatives using screening in vitro methods (erythrocyte hemolysis test and AChE inhibition test) and determined the effect of our new compounds on the interruption of the cycle of A549 cells (human lung adenocarcinoma). In addition, an assessment of the degree of differentiation of cells was tested. The division into early or late apoptotic cells and necrotic was specified as a function of the pro-apoptotic properties of tested benzimidazole derivatives.

## Results and discussion

### Benzimidazoles and their induction of the apoptosis in A549 cells

**Fig. 1:**
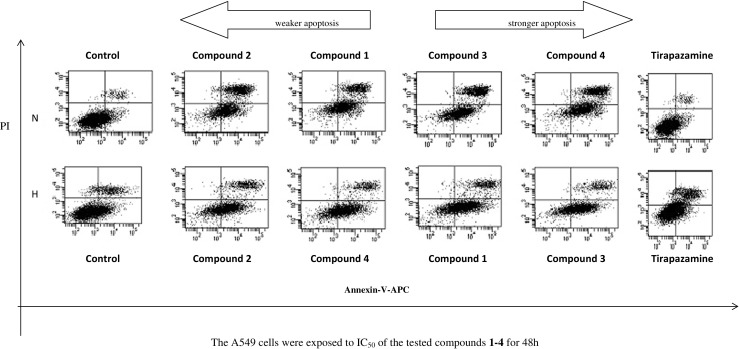
Results of Annexin V-PI test for normoxia (N) and hypoxia (H). The A549 cells were exposed to IC50 of the tested compounds **1**–**4** for 48 h

**Fig. 2:**
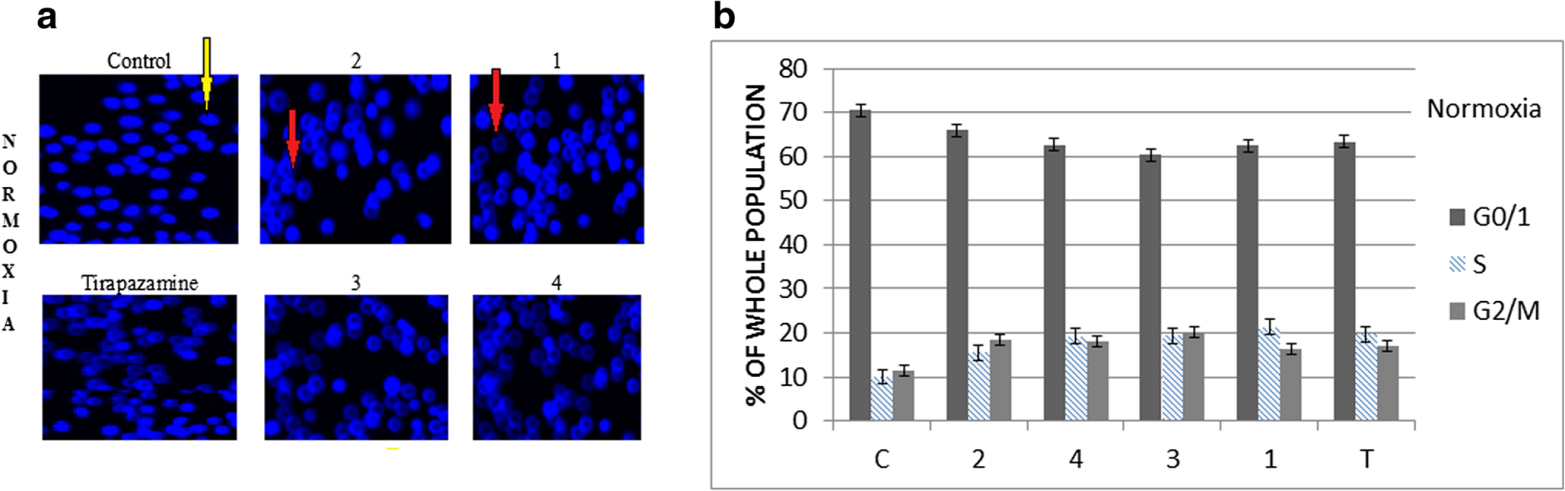
Visualization of apoptotic cells treated with the tested derivatives and tirapazamine in normoxia. The percentage of different cell populations identified by Hoechst (part A) and PI/Annexin V (part B) assay. *p < 0.05 (mean + SD; *n* = 3), *yellow arrows* indicate normal cells, and *red arrows* indicate apoptosis cells (*c* control, *T* tirapazamine, compounds **1**–**4**, *apo* apoptosis, *nec* necrosis**)**

**Fig. 3:**
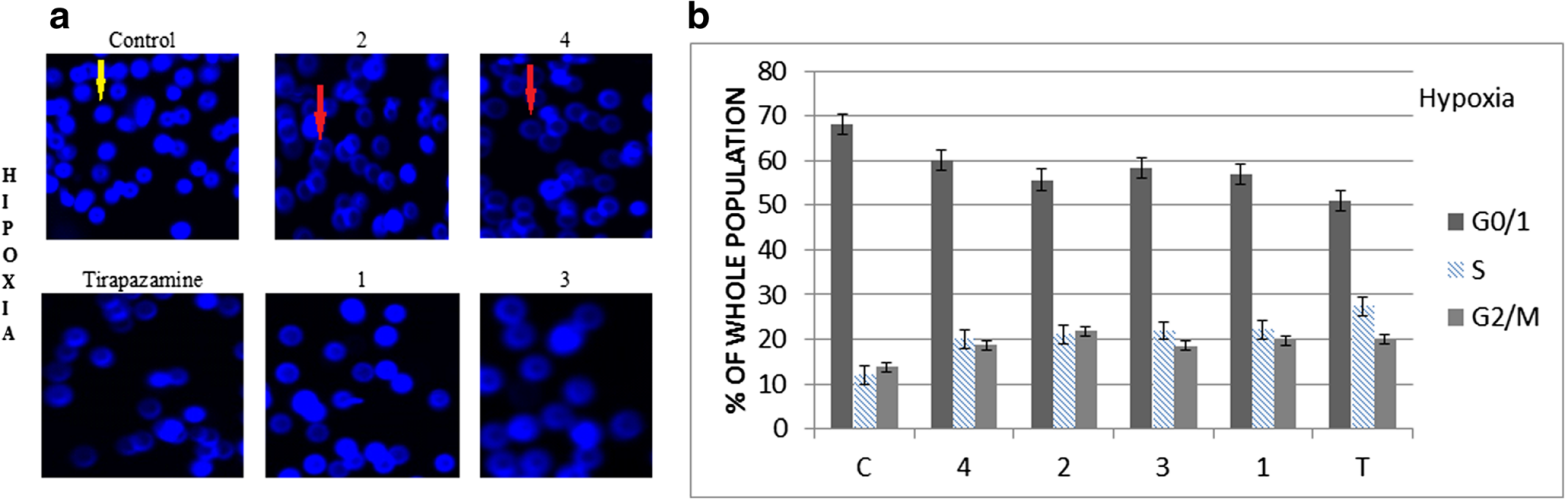
Visualization of apoptotic cells treated with the tested derivatives and tirapazamine in hypoxia. The percentage of different cell populations identified by Hoechst (part A) and PI/Annexin V (part B) assay. *p < 0.05 (mean + SD for three independent experiments; *n* = 3), *yellow arrows* indicate normal cells, and *red arrows* indicate apoptosis cells (*c* control, *T* tirapazamine, compounds **1**–**4**, *apo* apoptosis, *nec* necrosis)

Continuing studies on the cytotoxic activity of the new benzimidazole derivatives, the Annexin, and propidium iodide (PI) assay were carried out (Fig. 1). This analysis allowed determining the pathway of tumor cell death which occurs under the effect of the tested compounds. The participation of the alive cells and cells in early apoptosis and late apoptosis or necrosis in the population of A549 cells line was specified. The fundamental problem concerned the evaluation and comparison of the degree of induced apoptosis and necrosis. Within the conducted experiments (test Annexin V + PI), the dominance of early apoptotic cell population relating other cells (late apoptotic and necrotic) was observed, both in the culture performed in normoxia and hypoxia, in time up to 48 h (Figs. 2 and 3). In a series of samples treated with the tested compounds **1**–**4** under hypoxic conditions, the effect of severity of apoptosis cells was 60 % greater than in the control, while in normoxia, it was higher only up to 50 %. Generally, the results presented in Figs. 1, 2b, and 3b showed that hypoxia induced early apoptosis to a greater extent than normoxia for all tested substances. The strongest results proved compound **1** (*N*-oxide) and next compound **2** (analog of compound **1**) (Figs. 2 and 3 parts B). Comparing the induction of apoptosis under benzimidazole treatment with the induction of apoptosis under the influence of tirapazamine, it can be easily observed the beneficial pro-apoptotic effect of the tested compounds especially under hypoxic conditions (Figs. 2 and 3 parts B). The highest difference between the effect of inducing apoptosis in both series of the tested samples showed compound **3** (58/74 % N/H) (Figs. 2 and 3 parts B). The sequence of the other derivatives with the potency of induction of apoptosis in normoxia/ hypoxia is as follows: compound 1 > 2 > 4 (Figs. 2 and 3 parts B). The analysis confirms the selectivity of *N*-oxide (compounds **1**, **3**) under hypoxic conditions and determines that the derivatives having the substituent nitrophenyl (compounds **1**, **2**) are suitable in their apoptotic strength against the derivatives of substituent chlorophenyl (compounds **3**, **4**).

The results of Annexin V and PI test were also confirmed by the visualization of apoptotic cells in the analyzed samples (Figs. 2 and 3 parts A). In normoxia, we observed a clear effect of increasing the number of apoptotic cells compared to the control in all tested assays (Figs. 2 and 3 parts A). However, in hypoxia under the tested derivatives, the further progress of apoptosis (there were a few cells non-apoptotic visible in the field of vision) was seen and it led to necrosis which has been observed on the basis of a far smaller number of cells in the visual field (Figs. 2 and 3 parts A). It is worth noticing that the increase of necrotic cell growth is a result of a gradual cell death through apoptosis pathway and not through a strong, immediate cytotoxic action of compounds. The discussed results take into account the slight apoptosis and poorer necrosis in the control cells in the tested samples in both environments: normoxic and hypoxic.

### Benzimidazole and their influence on the cell cycle interruption

**Fig. 4:**
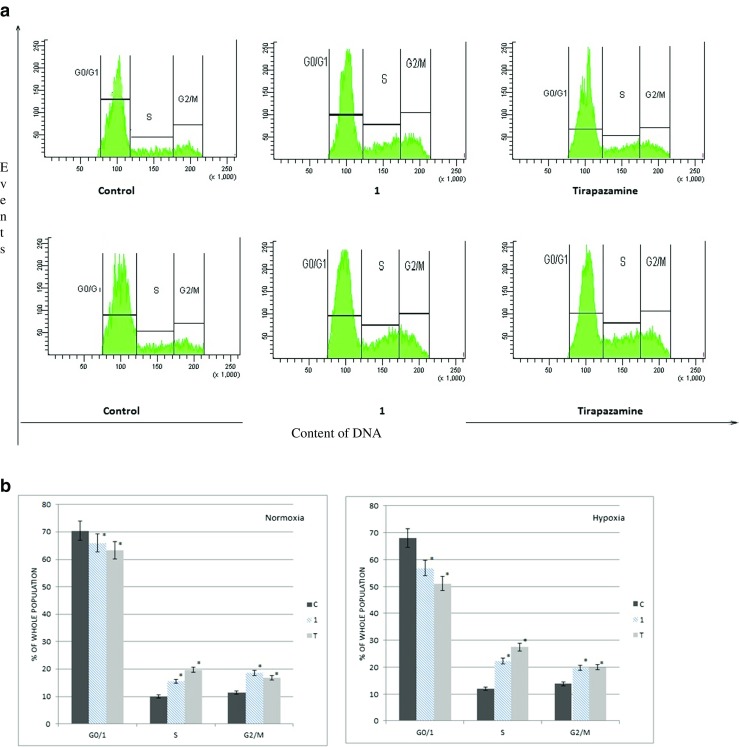
The percentage of cells in different phases of the cycle, after application of the compound **1** and tirapazamine in normoxia (N) and hypoxia (H). **a** Histogram of the flow cytometric DNA content for compound **1** analysis. **b** Cell cycle specification for compound **1** (*c* control, *T* tirapazamine). *p < 0.05 (mean + SD for three independent experiments; *n* = 3)

**Fig. 5:**
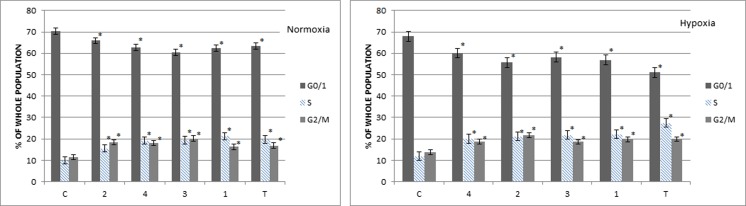
The percentage of cells in various phases of the cycle, after application of the compounds **1**–**4** and tirapazamine under normoxic and hypoxic conditions. Cell cycle specification for all tested compounds **1**–**4** (*c* control, *T* tirapazamine). *p* < 0.05 (mean + SD for three independent experiments; *n* = 3)

All tested compounds in normoxia and hypoxia had specific effects on the cell cycle of A549 cells, causing particularly the increase of the number of cells in the S phase replication, while the growth of cells in the G0/1 was inhibited. The largest increase of the number of cells in S phase was induced by the activity of compound **1**. Additionally, compound **1** more strongly inhibited the S phase in hypoxic conditions (the increase 2.2-fold) than in normoxic environment (the increase 1.5-fold) (Fig. 4). Other compounds **2**–**4** showed the specificity of inhibition of DNA synthesis in S phase in the conditions of culture as well. It should be noted that the N-oxide derivatives (compounds **1**, **3**) more strongly influence S phase under hypoxic conditions than normoxic. In addition, the substituent nitrophenyl affects higher activity of the benzimidazole derivatives than the chlorophenyl in both conditions. The consequence of the inhibition of DNA synthesis in S phase was the enhancement of the inhibition of the cell division in G2/M. All tests were performed in comparison with the reference compound tirapazamine, which the obtained results are consistent with previously published results and confirm the selectivity of them to the S phase of the cell cycle, particularly in hypoxia (Fig. 5) [14].

### Benzimidazoles and their lysis action

**Fig. 6:**
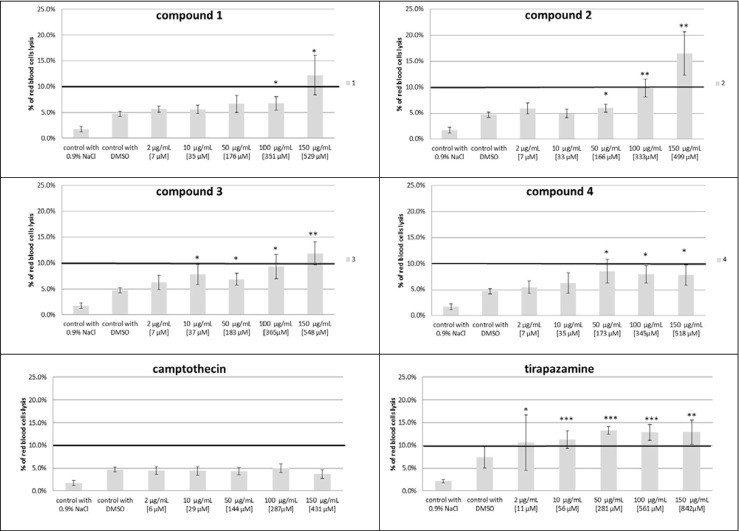
The percentage of red blood cell lysis obtained from the interaction compounds **1**–**4**, camptothecin, and tirapazamine with human erythrocytes, compared to the 100 % hemolysis; *n* = 5, *p = 0.05, **p = 0.06–0.001, ***p < 0.001 vs. control with DMSO

In this part of our experiments, the effect of four new compounds (**1**–**4**) and two standards (camptothecin and tirapazamine) on the human erythrocyte hemolysis was defined. We observed that the highest concentration of compounds **1**, **2**, and **3** slightly exceeded 10 % of hemolysis compared to positive control (Triton X-100 at 0.2 %). Ten percent of hemolysis is considered as the acceptable and potentially safe level for the integrity of cell membranes. Therefore, the compounds **1**, **2**, and **3** in the concentration range between 2 to 100 μg/mL can be considered as biocompatible. Compound **4** proved its biocompatible in all tested concentrations (% hemolysis does not exceed the value of 10). Tirapazamine already at the lowest tested concentration of 2 μg/mL caused hemolysis of greater than 10 %. The second reference substance camptothecin in the tested concentrations showed no adverse effect on the integrity of erythrocyte membranes. In this case, the designated % hemolysis of erythrocytes was not significantly different from the sample incubated with the solvent (DMSO) (Fig. 6).

**Fig. 7:**
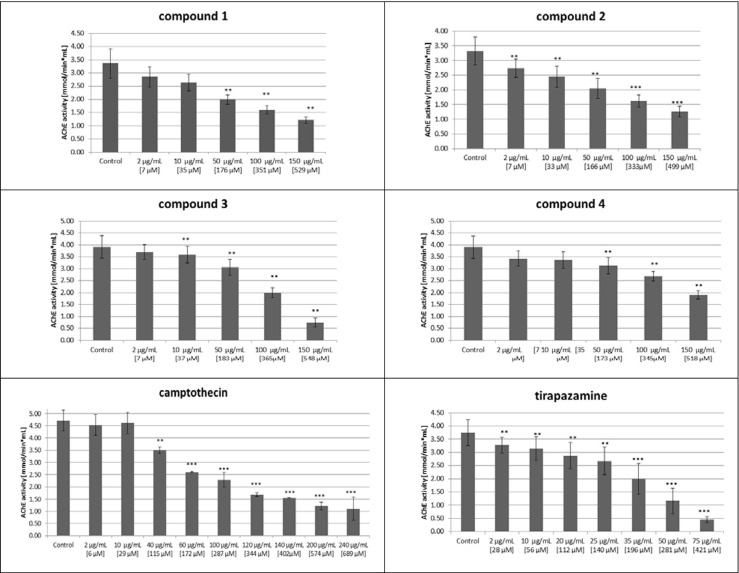
Influence of compounds **1**–**4**, camptothecin, and tirapazamine on human erythrocyte acetylcholinesterase activity. *n* = 5, **p = 0.06–0.001, ***p < 0.001 vs. control

**Fig. 8:**
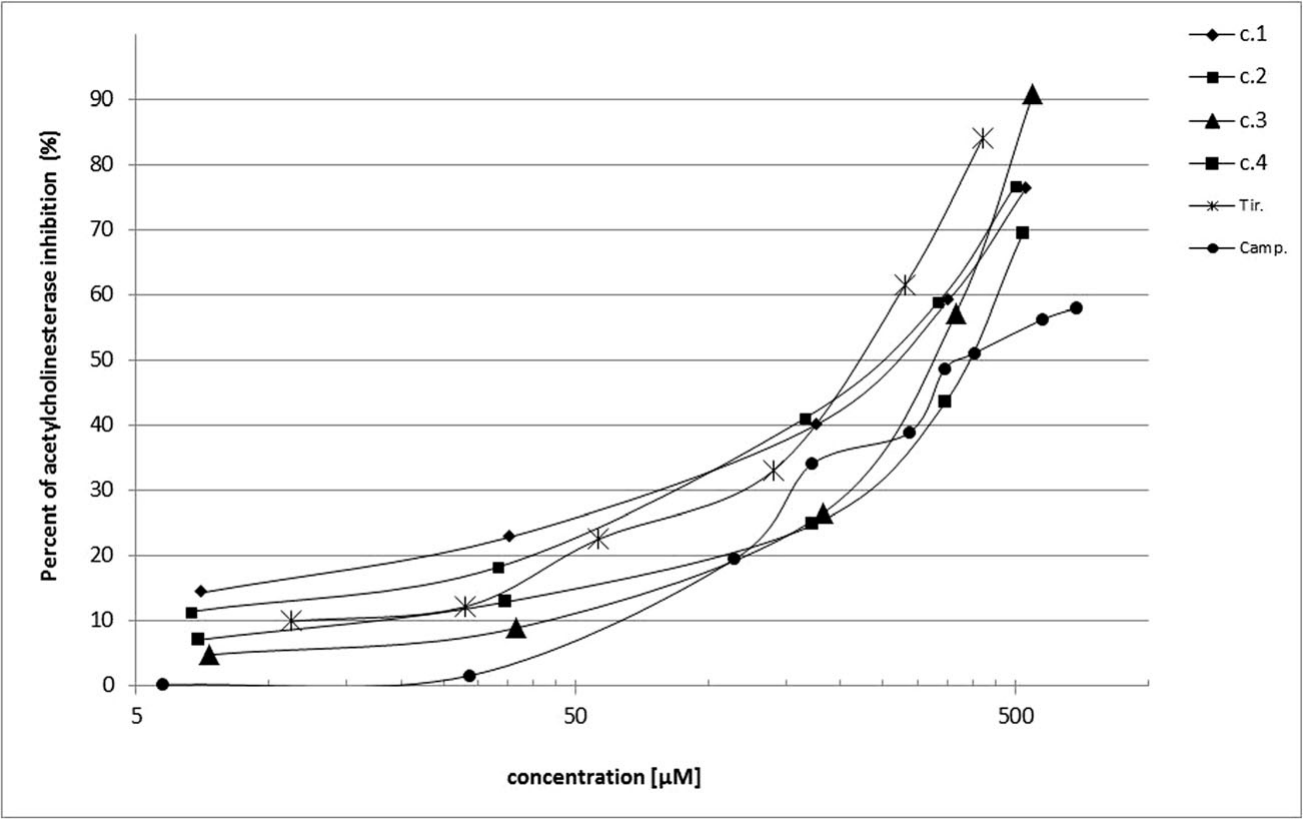
Inhibition of acetylcholinesterase activity by various concentrations of examined compounds (*c* compounds **1**–**4**, *Camp* camptothecin, *Tir* tirapazamine); *n* = 5

The next stage of our in vitro biological activity studies of tested compounds **1**–**4**, tirapazamine, and camptothecin toward inhibition of activity of human erythrocyte acetylcholinesterase was performed. As presented in Fig. 7, all compounds at the various concentrations contributed to the statistically significant decrease of AChE activity. Otherwise, tirapazamine in the whole range of used concentration (for example 75 μg/mL–0.4 mmol/min mL) exerted the strongest inhibition of AChE. Comparing IC_50_ values of compounds **1**, **2**, and **4** with IC_50_ value of used standard camptothecin (Table 2), the lack of statistical difference between them was observed. Only IC_50_ of compound **3** was statistically significantly higher which testifies to a much weaker impact of this compound on the enzyme (889.5 ± 26.2/399.0 ± 55.9, *p* = 0.01). Comparing IC_50_ values of all tested compounds with IC_50_ value of the next used standard tirapazamine, the much lower inhibition was watched. In this aspect, our new compounds as analogs of tirapazamine demonstrated potential lower undesirable action. We used the third standard tacrine as a typical AChE inhibitor. Regarding tacrine, our compounds possess negligible inhibitory activity on this enzyme (Fig. 8).

The dependence of acetylcholinesterase inhibition obtained during incubation of human erythrocytes of healthy volunteers with various concentrations of examined compounds, as well as IC_50_ values, is presented in Fig. 8 and Table 1. From these results, we can make the proposal of correlation between in vitro and in vivo action of the used new benzimidazoles. Both of these experiments confirmed their low potential inhibition of AChE enzyme.

**Table 1:** The IC_50_ values for activities toward human erythrocyte acetylcholinesterase (mean ± SD); *n* = 5; *p* values versus three standards (camptothecin, tirapazamine, and tacrine-non-competitive standards of AChE inhibition)

Compound	IC_50_ (μmol/L)	Comparison with IC_50_ of standards		
		Camptothecin	Tirapazamine	Tacrine
		399.0 ± 55.9	143.8 ± 53.8	0.239 ± 0.09
1	377.4 ± 59.4	NS; (*p* = 0.608)	*p* < 0.001	*p* < 0.001
2	372.9 + 59.4	NS; (*p* = 0.719)	*p* < 0.001	*p* < 0.001
3	889.5 ± 26.2	*p* = 0.01	*p* < 0.001	*p* < 0.001
4	306.8 ± 48.2	NS; ( *p* = 0.06)	*p* < 0.001	*p* < 0.001

## Experimental section

### Tested compounds

The activity and the effect on the life processes of tumor cell line A549 were related to new benzimidazole derivatives and their analogs with *N*-oxide bond. The experiments were carried out according to standard test procedures and based on observations and modifications of the performed tests. The used compounds were prepared according to the procedure described earlier [12]. The concentration of the tested compounds corresponded to the previously designated IC_50_ values [13]. Tirapazamine has been used as a reference compound due to its unique pro-apoptotic properties and high selectivity to hypoxic conditions. Compounds were identified by numbers from **1** to **4**, respectively (Table 2).

**Table 2:**
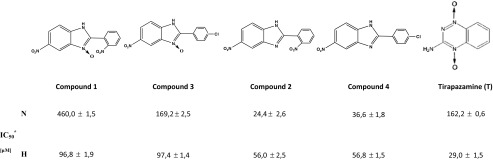
The structures of the tested substances and their IC_50_ values on A549 cell line in normoxia (N) and hypoxia (H)

^a^Data are expressed as the mean ± SD, *n* = 3

### Cell culture

A549 cells— a human lung adenocarcinoma A549 cell line— were purchased from the Health Protection Agency Culture Collections (ECACC, Salisburgy, UK), cultured in F12K medium (HyClone, Loughborough, UK) supplemented with 10 % heat-inactivated fetal bovine serum (FBS) (Lonza, Basel, Switzerland), penicillin (10,000 U/mL), and streptomycin (10,000 μg/mL) in 5 % CO_2_ at 37 °C. Hypoxic cells were obtained by culture of A549 cells in a hypoxic incubator in 1 % O_2_ and 5 % CO_2_ at 37 °C. The incubation at both conditions was conducted for 24 h before treatment.

Morphological changes of the normoxic and hypoxic cells as an effect of the activity of the tested compounds were evaluated with a phase-contrast microscope at ×100 magnification (OptaTech, Warsaw, Poland).

The statistical data are expressed as mean ± SD, by using Student’s *t* test, *n* = 3 for each independent experiment. *p < 0.01 was considered significant.

#### Assessment of apoptosis and flow cytometry analysis

In T25 flasks, 5 ∗ 10^5^ A549 cells were seeded for 24 h and treated with benzimidazoles and tirapazamine for further 48 h. The phosphate-buffered saline (PBS) was used for washing, and the acutase was used for detaching. The detached cells were diluted in buffer (100 μL/1 ∗ 10^5^cells) containing APC-conjugated Annexin V/ PI (4 μL/1 ∗ 10^5^cells). Next, the incubation was conducted for 20 min at dark area and room temperature. After that time, the flow cytometry analysis was carried out (FACS Canto II, Becton Dickinson, USA). The apoptosis and necrosis populations were assessed by PI versus Annexin V correlation. The cells stained with Annexin V only were recognized as early apoptosis, and the Annexin V and PI double-stained cells were recognized as late apoptosis or necrosis.

#### Confocal visualization of apoptotic cells

In T25 flasks, 5 ∗ 10^5^ A549 cells were seeded for 24 h and treated with benzimidazoles and tirapazamine for further 48 h, obtaining 80 % confluence of cell population. The PBS was used for washing, and the acutase was used for culturing. Next, the detached cells were washed and permeabilized by 70 % methanol at −21 °C. To the sample was added the optimized amount of Hoechst 33342 (Thermo Scientific, Warsaw, Poland) reagent at concentration 1 μg/mL. After 20 min of incubation at room temperature, the positively stained population (dark-blue cells) was observed using confocal microscopy at ×100 magnification (Olympus bx61vs). The percentage of different cell populations was identified by Hoechst (part A) and PI/Annexin V (part B) assay. *p < 0.05 (mean + SD; *n* = 3), yellow arrows indicate normal cells, and red arrows indicate apoptosis cells.

#### Measurement of DNA content by flow cytometry analysis

To tested compounds and control sample as well, 5 ∗ 10^5^ A549 cells were exhibited and collected by using acutase. The cells were washed with PBS and fixed in 70 % ethanol. Next, the incubation with RNase (100 μg/mL, Sigma-Aldrich, Munich, Germany) and PI (50 μg/mL, Sigma-Aldrich, Munich, Germany) was carried out for 30 min at dark space at room temperature. The data from above 10,000 cells of each sample were scanned by FACS Scan Canto II (Becton Dickinson, USA), and the amount of DNA was analyzed.

### Toxicity assay

#### Reagents

We used 0.9 % physiological sodium chloride solution (0.15 mol/L) and 0.1 mol/L phosphate buffer (pH = 7.0 and pH = 8.0) prepared from reagents obtained from Polish Chemical Reagents (Gliwice, Poland). The 5,5′-dithiobisnitrobenzoic acid (DTNB) and acetylthiocholine iodide (enzyme substrate) were obtained from Sigma-Aldrich (Munich, Germany). Exactly before the experiments, DTNB (0.1 mol/L) with phosphate buffer pH = 7.0 and acetylthiocholine iodide (21.67 mg/mL) with distilled water were diluted. Triton X-100C was obtained from Polish Chemical Reagents (Poland). Tacrine (Sigma-Aldrich, Munich, Germany) was used as a standard non-competitive inhibitor of acetylcholinesterase.

#### Materials

Blood samples were taken from healthy donors using a vacuum tube with potassium versenate. Collection was taken at the Regional Blood Donation and Hemotherapy Centre, Lodz. Erythrocytes were separated from the plasma and leucocytes by centrifugation (3000×*g*, 10 min) at 4 °C and washed three times with 0.9 % saline. The hemolyzed human erythrocytes for acetylcholinesterase (AChE) activity determination were used. They were stored at −30 °C until the measurements were taken. Immediately prior to each measurement, the hemolyzed erythrocytes were restored at 37 °C for 30 min. Once thawed, hemolyzed erythrocytes were not frozen again and were not used for retesting.

#### Methods

Activity of human erythrocyte acetylcholinesterase (AChE) was defined spectrophotometrically according to the Ellman [17] method with some modifications [18]. The freshly thawed erythrocytes in phosphate buffer (pH 8) were diluted (1:400). The 0.960 mL of this solution, 0.010 mL of DTNB solution (1 μmol/mL), and 0.020 mL of examined compound at concentration ranging from 2 to 150 μg/mL or appropriate volume of diluent were added to the cuvette and incubated for 5 min at 37 °C. To start the enzymatic reaction, 0.010 mL of acetylthiocholine iodide (0.75 μmol/L) was added. The continuous measurements of absorbance were performed at a wavelength of *λ* = 436 nm by means of a spectrophotometer (Cecil CE2021, London, England) at 37 °C for 6 min. The results were saved and analyzed with DATA STREAM CE3000 5.0 software. The activity of human erythrocyte AChE was calculated automatically, according to equation *E* = Δ Abs / *F* and expressed as [μg/min mL], where *E*—activity of AChE, Δ *A*—velocity of reaction [OD/min], and *F*—factor (*F* = dilution/absorption coefficient for the [TNB-]). The absorption coefficient for the [TNB^−^] ion in assay conditions was 10.6 mM^−1^ cm^−1^. AChE inhibition, expressed in %, was calculated using the equation ((*A*_0_ − *A*_IC_) ∗ 100 %) / *A*_0_, where *A*_0_ means AChE activity in the control sample, expressed as the rate of the enzymatic velocity [A/min], and *A*_IC_—AChE activity in the sample of examined compounds or reference standard, expressed, as above, as the rate of the enzymatic reaction [OD/min]. The IC_50_ value, defined as the drug concentration that inhibits 50 % AChE activity, was determined from a fitted sigmoidal concentration–response curve.

### Statistics analysis

All values of measured parameters were expressed as means ± SD. The Q-Dixon test to reject the results unreliable was used. Statistical tests were performed using a commercial software package (Statistica 10.0, StatSoft). The Shapiro–Wilk test was applied to determine whether the continuous variables were normally distributed. The *t* test was used to compare variables which showed normal distributions, while the Mann-Whitney test was used for variables showing non-normal distributions. A *p* value of less than 0.05 was considered as statistically significant.

#### Assay of red blood cell lysis

Assay of red blood cell lysis according to the early described method was performed [19]. Briefly, red blood cells at concentration of 2 % were incubated at 37 °C with compounds at concentration ranging from 2 to 150 μg/mL. After 1 h of incubation, the samples were centrifuged at 3000 rpm for 10 min and the absorbance of the supernatant was measured at 550 nm. Hemolysis of RBCs was expressed as percentage of released hemoglobin. A solution of RBCs incubated with Triton X-100 (0.2 % *v*/*v*) was used for determination of 100 % of hemoglobin release. A control with diluent (water or DMSO) was used to determine spontaneous hemolysis of RBCs and to compare it with hemolysis caused by investigated substances.

## Conclusions

Performing further analysis of the biological activity of the new benzimidazole derivatives with potential antitumor activity, the effect of these compounds on the disintegration of the cells in the cascade of pathological events of pro-apoptosis through apoptosis to necrosis as a phenomenon leading to the gradual but effective cancer cell death was determined. The performed tests showed pro-apoptotic properties of the tested compounds related to their ability to induce significant apoptosis in both normoxia and hypoxia conditions and the immediate elimination of the undesirable necrosis. These results confirm the preferred biological parameters of new benzimidazole derivatives especially in the context of the searching for new cytotoxic compounds from the group of selective anticancer agents. Our research conducted in normoxia and hypoxia proved the stability of these properties and clearly indicated the structure of the *N*-oxide as the factor ensuring the selectivity in hypoxia. With regard to the reference compound, the level of activity of all tested compounds is high, which demonstrates the accuracy of the selection of the studied structure with the characteristic substituents of the benzimidazole ring.

All tested benzimidazole derivatives also showed the DNA damaging potential. Because of their action on the cell cycle, they stopped the replication in S phase. By this property, on the one hand, they allow efficient transition of cells to a controlled way of death. On the other hand, they more strongly inhibit the topoisomerase I activity [15, 16] at the same time, and consequently, they promote damage to the genetic material of tumor cells. In comparison to the reference compound, *N*-oxide 2-(2-nitrophenyl)-1*H*-benzimidazole (compound **1**) possesses the greater degree of action on the cell cycle in hypoxia. Probably, it is due to the presence of N-oxide function of its structure, because such studied analogs showed higher activity with respect to derivatives without these groups. This conclusion is derived from the fact (what we observed during our experiments) that the increase of the activity of derivatives of benzimidazole is correlated with the presence of the *N*-oxide bond. The analogous situation concerns derivatives with nitro group (the nitrophenyl substituent). They possess the higher activity relative to the derivatives with chloro group (the chlorophenyl substituent). In comparison to all other tested derivatives of benzimidazole and also relative to the reference compound, the most active in the context of the impact on the life processes of tumor cells is *N*-oxide 2-(2-nitrophenyl)-1*H*-benzimidazole (compound **1**). First of all, it was characterized by the greatest specificity to hypoxic conditions. Satisfactory results were also achieved for its analog, not having the structure of *N*-oxide (compound **2**). The study of benzimidazole biological activity should be continued especially as an aspect of broadening cell line types. This would allow evaluating their features against different types of tumors.

In this study, biocompatibility of the newly synthesized compounds and standards by means of the assay of the erythrocyte lysis was assessed as well. According to previous studies, less than 10 % of hemolysis is not considered cytotoxic [19]. Comparing the effects of the tested compounds and tirapazamine (standard substance) on the integrity of the erythrocyte membrane, it can be concluded that the newly synthesized compounds have a less adverse effect on the membrane of the erythrocytes. Tirapazamine already at the lowest test concentration of 2 μg/mL caused greater than 10 % hemolysis. The second reference substance camptothecin in the tested concentrations showed no adverse effect on the integrity of erythrocyte membranes. In this case, the designated % hemolysis of erythrocytes was not significantly different from the sample incubated with the solvent (DMSO). The erythrotoxicity test results indicate that the tested newly synthesized compounds can be considered potentially biocompatible, which can make a positive contribution for further testing of these compounds in animals.

In the next stage of the study, the effect of new benzimidazole derivatives on the activity of human erythrocyte AChE was conducted. The tested compounds were designed as inhibitors of topoisomerase I [13]. From literature, it is known that camptothecin and other inhibitors of topoisomerase I also tend to result in the inhibition of AChE [20, 21]. Using them can cause the cholinergic syndrome, usually mild, although this is one of the main reasons for cancelling such drugs from the treatment in the anticancer therapy. [17]. In our studies, we used the adapted in vitro testing of the new compounds, i.e., the method of Ellman [17]. It is the reference method used in the laboratory diagnosis of acute poisonings among other organophosphorus pesticides that are potent inhibitors of AChE. This method is also used in vitro and in vivo assays to assess the efficacy of oxime for the reactivation of AChE.

The AChE is an essential enzyme primarily expressed in the central and peripheral nervous systems. However, different isoforms of AChE are also constitutive of various cell types, e.g., human fibroblasts, osteoblasts, kidney cells, erythrocytes, vascular endothelial cells, and leukocytes, as well as lung, and spleen cells. It is well established that the biological role of AChE is not limited to its classical role in hydrolyzing acetylcholine. In many kinds of tissue, AChE plays non-classical functions such as the influence on apoptotic sensitivity, cellular proliferation, and differentiation, suggesting a possible role of cholinesterases in tumorigenesis and stress responses related to inflammation. Furthermore, abnormal expression and structural alteration of AChE and multiple activities have been found in different types of tumors, such as brain, lung, ovarian, breast, hepatocellular, renal, and colon cancers, which indicate the involvement of AChE in regulating the tumor development. However, the role of AChE in tumor progression remains unclear [19, 20, 21].

Our research has shown that all tested compounds with different strengths inhibit the enzymatic activity of human erythrocyte AChE. The experiments showed that the weakest substance is compound **3** (it has got statistically significantly higher IC_50_ value compared with camptothecin). IC_50_ values for compounds **1**, **2**, and **4** did not differ significantly from the value of camptothecin IC_50_ (standard), which may indicate comparable tendency to induce a side effect. In literature, there is no research on the effects of tirapazamine on the activity of AChE. What is interesting, tirapazamine proved its strongest power according to all tested compounds—its IC_50_ was statistically lower than of all other tested compounds. Currently, the third phase of clinical trials of this compound is ongoing [22]. It is known that in addition to neutropenia, one of the most frequently reported symptoms of toxicity occurring in the approach of the anticancer therapy is nausea, vomiting, and diarrhea of unknown etiology. These are non-specific symptoms, but they can appear as a result of excessive inhibition of AChE by tirapazamine as well [17].

However, it is worth emphasizing that the inhibition of AChE observed in our study is not particularly strong. In order to compare, the IC_50_ value was determined for tacrine, the strong typical inhibitor of AChE activity. The IC_50_ value of tacrine was statistically significantly lower than for all the tested compounds.
